# DECOLONIZING GERONTOLOGY BY REPATRIATING EXPERTISE: THEORY, RESEARCH DESIGN, AND A RESEARCH STUDY EXAMPLE

**DOI:** 10.1093/geroni/igae098.4382

**Published:** 2024-12-31

**Authors:** Alexandra Crampton

**Affiliations:** Marquette University, Milwaukee, Wisconsin, United States

## Abstract

This presentation provides a rationale, a research design, and a research example of decolonizing gerontology through integration of Indigenous philosophy and practices. Colonization in gerontology is identified on three levels; reducing complexity of aging into problems for outside experts to solve; associating aging with loss that negates (and potentially threatens) modern values of youth, independence, and productivity; and limiting the research role of older adults to that of consenting research subjects. Decolonization in gerontology explores complexity of lived experience, and expands the research role of older adults to actively challenge, deepen, and redirect the research. My research design incorporates Shawn Wilson’s decolonization work and lessons from the successful repatriation of Ahayu: da by Zuni negotiators. My research example is from a study conducted between July 2021 and July 2024 in a Midwestern retirement community where the average age is 86. Covid restrictions persisted through May, 2023. My research goal was to repatriate gerontological constructs, such as “successful aging” and “older adulthood” through community-engaged research. Data were collected using ethnographic methods of participant observation, in-depth interviews, archival document collection, and frequent consultation with research participants. Although “successful aging” and “older adulthood” were recognized by research participants, these constructs were rejected as non-informative by those confronting major disruption to roles, status, and/or functioning. Construct repatriation included reformulating aging as reckoning with loss of capacities, relationships, and other sources of meaning that had been integral to a sense of self and means of flourishing. Research presentations became a means of shared reckoning.

